# An Exon Signature to Estimate the Tumor Mutational Burden of Right-sided Colon Cancer Patients

**DOI:** 10.7150/jca.34363

**Published:** 2020-01-01

**Authors:** Wenbing Guo, Yelin Fu, Liangliang Jin, Kai Song, Ruihan Yu, Tianhao Li, Lishuang Qi, Yunyan Gu, Wenyuan Zhao, Zheng Guo

**Affiliations:** 1 College of Bioinformatics Science and Technology, Harbin Medical University, Harbin, 150086, China; Phone: (86 451) 8661-5933; Fax: (86 451) 8666-9617; 2 Department of Bioinformatics, Key Laboratory of Ministry of Education for Gastrointestinal Cancer, School of Basic Medical Sciences, Fujian Medical University, Fuzhou, 350122, China; 3 Key Laboratory of Medical Bioinformatics, Fujian Province, Fuzhou 350122, China

**Keywords:** tumor mutational burden, the right-sided colon cancer, the coding DNA sequences, a cancer-specific signature

## Abstract

The clinical applicability of the whole-exome sequencing (WES) in estimating tumor mutational burden (TMB) is currently limited by high cost, time-consuming and tissue availability. And given to the differences in the mutational landscapes among different types of cancer, we aimed to develop a cancer-specific signature to estimate TMB for right-sided colon cancer patients (RCC). Using WES data of 315 RCC patients, we identified the exons in which the number of mutational sites of the coding DNA sequences associated with TMB through linear regression analysis. Then, among these exons, we extracted a signature composed by 102 exons (~0.13 Mbp) through a heuristic selection procedure. The TMB estimated by the signature was highly correlated with those calculated by WES in the discovery dataset (R^2^=0.9869) and three independent validation datasets (R^2^=0.9351, R^2^=0.8063 and R^2^=0.9527, respectively). And the performance of the signature was superior to a colorectal-specific TMB estimation model contained 22 genes (~0.24 Mbp). Moreover, between TMB-high and TMB-low RCC patients, there were significantly differences in the frequencies of microsatellite instability status, CpG island methylator phenotype, *BRAF*, *KRAS* and *POLE*/*POLD1* mutation status (*p*<0.01). However, the performances of the signature in other types of cancer were dramatically degraded (left-sided colon cancer, R^2^=0.7849 and 0.9407, respectively; rectum, R^2^=0.5955 and R^2^=0.965, respectively; breast cancer, R^2^=0.8444; lung cancer, R^2^=0.5963), suggesting that it was necessary to develop cancer-specific TMB estimated signatures to estimate precisely the TMB in different types of cancer. In summary, we developed an exon signature that can accurately estimate TMB in RCC patients, and the cost and time required for the assessment of TMB can be considerably decreased, making it more suitable for blood and/or biopsy samples.

## Introduction

Colorectal cancer is one of the most commonly diagnosed cancers worldwide. The incidence is about 1.2 million per annum, and more than 600,000 patients die from this cancer every year 1, 2. Currently, cancers originating from proximal/distal to the splenic flexure are classified as right/left-sided colon cancer (RCC/LCC). RCC tumors derive from the embryonic midgut, whereas LCC tumors derive from embryonic hindgut 3. The different origins consequently contribute to tumors with a different gene expression and mutation profile. RCC patients are reported to be a higher incidence of *BRAF*, *POLE/POLD1* mutation, CIMP, MSI and genome hypermutation 4-8. Conversely, LCC tumors are characterized by higher frequency of *KRAS* mutation and chromosomal instability 9. These differences result in different prognoses for the two tumor types, and RCC tumors are associated with poorer patient outcome 3, 8, 9.

In recent years, immune checkpoint inhibitor therapy has shown great promise as a treatment for several cancers 10-12, and a few trials employed immunohistochemical (IHC) staining of PD-L1 (programmed death-ligand 1) on tumor cells and/or immune cells as a predictive biomarker to separate responders from non-responders 13, 14. However, there is accumulating evidence that the discriminatory power of PD-L1 expression has limitations 15, 16. Alternatively, another emerging biomarker for response to immunotherapy is the overall number of mutations presented in a tumor specimen, termed as the tumor mutational load or tumor mutational burden (TMB). Indeed, the patients with highly TMB are more likely to harbor neoantigens, which makes them tend to benefit from immune checkpoint blockades 10, 17, 18. Therefore, a refined assessment of TMB is critical for informing treatment recommendations.

Currently, whole-exome sequencing (WES) is a primary method to estimate TMB levels. And the TMB levels were divided into two groups according to the numbers of somatic mutation per megabase (Mbp) of genome coding area: low (<20 mut/Mbp) and high (≥20 mut/Mbp) 19, 20. However, due to the infrastructure requirements, high cost, substantial turnaround time and excessive information about variants/genes of unknown significance, WES is not yet routinely available in the clinical practices 21, 22. In contrast, next-generation sequencing (NGS) panels composed by ~200-600 oncogenes, tumor suppressor genes, and members of pathways deemed actionable by targeted therapies, such as FoundationOne panel 23, 24, UW-OncoPlex panel 25 and MSK-IMPACT panel 26, 27, are widely used to investigate the TMB levels of tumors nowadays. However, lacking of prioritization, those NGS panels that consist of genes known or suspected to be relevant to cancer may not perform better than expected by chance. And the cost of them with more than 200 genes is still high, which may be limited for the routine molecular diagnostics, especially for blood and/or biopsy specimens. More importantly, most of the current panels are derived from multiple types of tumor patients 23, 24, 26, 27, but on account of the considerable differences in mutational landscapes among different types of cancer, a cancer-specific estimation panel is necessary to estimate precisely TMB for a specific type of cancer. Recently, Lyu et al. has constructed a cancer-specific TMB estimated model, which was composed of 22 genes, for colorectal cancer 28. However, it is fairly inconvenient to clinical practice because of the large targeted sequencing territory and complex parameters.

Therefore, in this study, we sought to develop a more cost-effective and clinically available signature to accurately predict the TMB of colon patients based on the coding DNA sequences (CDS). And given that the patients with RCC may be more sensitive to immunotherapy because of higher TMB-high rate compared to LCC patients 29, 30, we mainly concentrated on the RCC. The cancer-specific signature may allow the design of customized panels for the targeted sequencing of selected genome regions, instead of WES, to estimate TMB, decreasing the cost and time required for the assessment of mutational burden.

## Material and methods

### Data sources and preprocessing

The WES mutational data was collected from the cBioPortal(http://www.cbioportal.org/data_sets.jsp) and The Cancer Genome Atlas (TCGA, https://portal.gdc.cancer.gov/) databases. All datasets were described in detail in Table 1. The 315 RCC samples published by Giannakis et al. 31 were used for the construction of the exon signature. The WES somatic mutational data from three independent studies (n=225 for TCGA; n=57 for Vasaikar et al. and n=72 for Seshagiri et al.) 32, 33 were retrieved to test the performances of the exon signature. Notably, there were no specific location information for patients in the Seshagiri dataset to distinguish RCC and LCC. Meanwhile, the LCC, rectum, breast cancer and lung cancer samples showed in Table 1 were utilized to investigate whether the exon signature trained using RCC samples can also be employed to estimate the TMB for patients with other cancer types.

The human reference genome (hg19 GRCh37 and hg38 GRCh38) were downloaded from UCSC Genome Browser (http://genome.ucsc.edu/cgi-bin/hgTables). Since there were many gene transcripts for every gene, the CDS length of the longest transcript was selected for the corresponding gene.

The TMB for each sample was calculated by measuring the total of somatic mutations occurring in the CDS regions in the sequenced gene. Of note, synonymous mutations were counted as well to reduce sampling noise and as an attempt to capture mutational processes contributing to neoantigen 23, 34. To normalized against mutations per megabase, we divided the number of somatic mutations by the total genomic territory sequenced 23, 35.

### Performance evaluation of the existing gene panels

The MSK-IMPACT (n=341) 26 and FoundationOne (n=315) gene panel 24, 36, which developed from non-cancer-specific patients and contained all exons in the corresponding genes and were comparatively mature, are widely used in routine molecular diagnostics nowadays 36, 37. Here, we adopted a stratified randomized resampling procedure to investigate whether the performances of the MSK-IMPACT (or FoundationOne) panel was significantly different from randomly selected panels with the same number of genes. The stratified randomized resampling procedure may ensure that the length of the randomly selected set was close to that of the MSK-IMPACT (or FoundationOne) panel.

We firstly stratified all WES genes into 100 subsets according to the CDS length of every gene and counted the number of genes of MSK-IMPACT (or FoundationOne) panel in each subset. Subsequently, we resampled the same number of genes as that of MSK-IMPACT (or FoundationOne) panel in the corresponding subset and recorded the randomly selected gene set. Then all the selected sets formed a random panel. The procedure was repeated 1000 times, resulting in 1000 random panels. For each random panel, the R^2^ between estimated and WES TMB was calculated by linear regression analysis. Finally, we compared the average R^2^ that measured by randomly selected panels with the actual R^2^ calculated by the MSK-IMPACT (or FoundationOne) gene panel.

### Development of the exon signature for approximating TMB

For every exon, we determined whether the mutational values within the samples were associated with WES TMB by linear regression analysis. And then, the *p*-value was subjected to Benjamini-Hochberg multiple testing correction 38, and regions with false discovery rate (FDR)≤0.01 were taken as candidate exons for the following analysis.

Next, based on the candidate exons, we applied a heuristic selection procedure to search a signature that achieved the maximum R^2^ value for estimating TMB in RCC samples. The candidate exon with maximal R^2^ was chosen as the seed and then added another candidate exon to a set one at a time until the R^2^ did not increase. Notably, when added an exon to the set, we also re-considered if there were better combinations in the set and deleted the exon that cannot improve R^2^. Finally, a set of exons with the maximal R^2^ was chosen as an estimated signature for TMB in RCC samples.

### Statistical analysis

A two-tailed Fisher's exact test was applied to estimate the molecular differences between TMB-high and TMB-low groups, such as MSI, CIMP phenotype, *KRAS*, *BRAF* and *POLE/POLD1* mutational status. And linear regression analysis were used to determine the consistency between the estimated TMB and WES TMB. All statistical analyses were performed by using the R software package version 3.4.2.

## Results

### Evaluation of the non-cancer-specific gene panels

**Figure 1:**
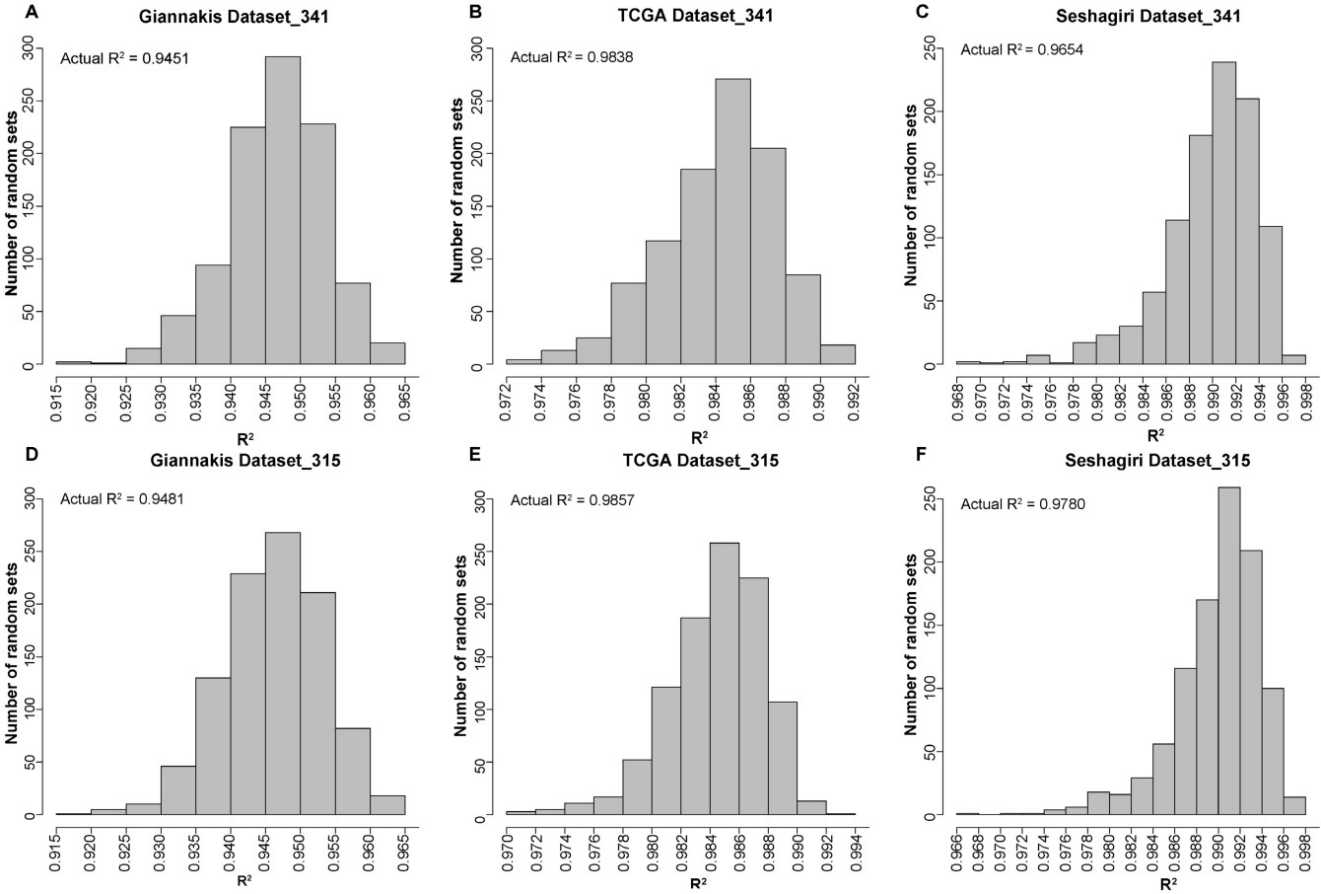
** Performance evaluation of the existing gene panels through 1000 times stratified randomized resampling procedure.** (A-D) Empirical distribution of R^2^ between the estimated TMB and WES TMB for the randomly selected panels composed of 341 genes in Giannakis (A), TCGA (B) and Vasaikar (C) dataste, respectively. (D-F) Empirical distribution of R^2^ between the estimated TMB and WES TMB for the randomly selected panels composed of 315 genes in Giannakis (D), TCGA (E) and Vasaikar (F) dataste, respectively.

We firstly applied the MSK-IMPACT panel with all coding exons from 341 genes to 315 RCC samples in the Giannakis WES dataset and found that the estimated TMB was highly correlated with that observed in WES (linear regression analysis, R^2^=0.9451). Then, randomly selected panels with the same number of genes as the MSK-IMPACT panel were structured through a stratified resampling procedure (see methods). After the procedure was repeated 1000 times, we found that the average R^2^ measured by the randomly selected panels was 0.9465, which was not significantly different from that estimated by the MSK-IMPACT panel (R^2^=0.9451) (**Figure 1A**). Similarly, in the TCGA and Seshagiri datasets, we also demonstrated that measurements of TMB by the MSK-IMPACT panel were strongly reflective of measurements from WES (TCGA dataset, R^2^=0.9838; Seshagiri dataset, R^2^=0.9654), but the R^2^ of the panel was not dramatically different from that measured by the randomly selected panels as well (TCGA dataset, average R^2^=0.9842, **Figure 1B**; Seshagiri dataset, average R^2^=0.9887, **Figure 1C**). Similar results were observed for the FoundationOne gene panel (n=315) in these three WES datasets (**Figure 1D-F**).

In summary, the above results showed that these two non-cancer-specific gene panels perform similarly to randomly selected panels with the same number of genes, and additional signature for approximating the TMB was needed.

### Identification and validation of the exon signature for estimating TMB

**Figure 2:**
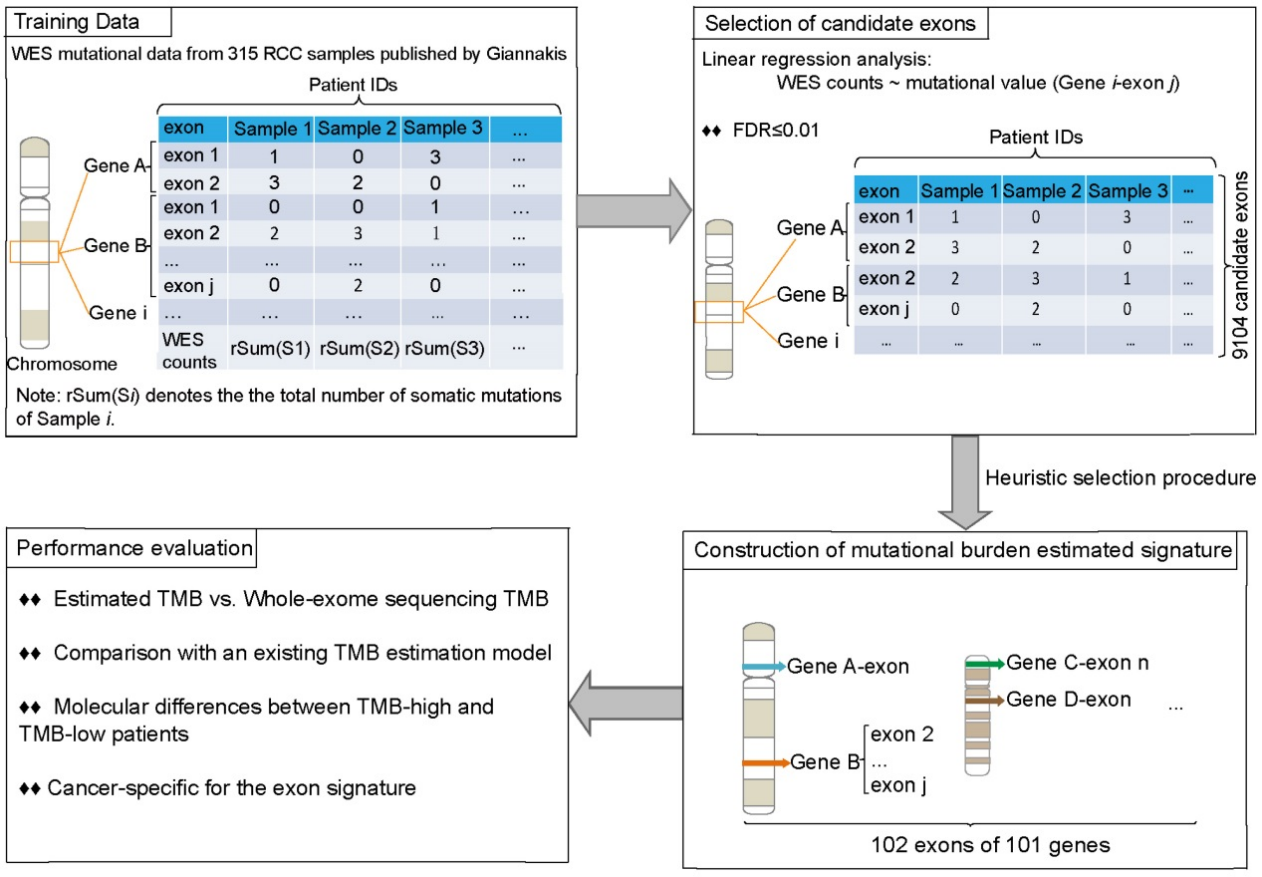
The process of the development and analysis of the exon signature for patients with RCC.

**Figure 3:**
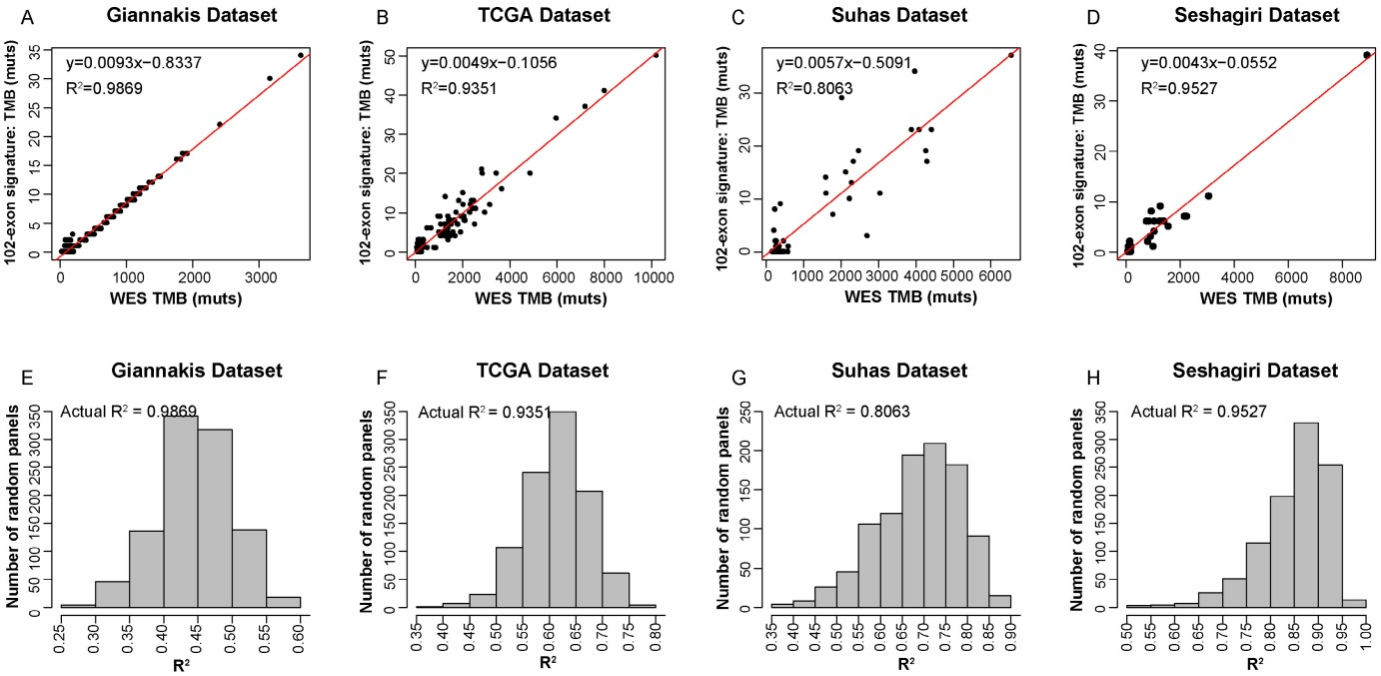
** Precision of TMB estimation for the 102-exon signature.** (A-D) Estimated TMB vs. WES TMB in Giannakis (A), TCGA (B), Vasaikar (C) and Seshagiri (D) dataste, respectively. (E-H) Empirical distribution of R^2^ between the TMB estimated by the exon-sginature and WES TMB for the randomly selected panels composed of 102 exon in Giannakis (E), TCGA (F), Vasaikar (G) and Seshagiri (H) dataste, respectively.

Figure 2 described the flowchart for identifying and validating the exon signature. For each exon, we assessed the association of its mutations with WES TMB by linear regression analysis. With FDR≤0.01, we identified 9104 candidate exons in which the number of mutational sites of the CDS regions were significantly associated with TMB in the 315 RCC samples in Giannakis WES dataset. Then, we took the exon with maximal R^2^ as a seed and utilized a heuristic selection procedure to identify a signature for estimating the TMB (see methods). Finally, 102 exons of 101 genes were extracted as the signature (Supplementary Table 1), termed as 102-exon signature, and the estimated TMB was highly correlated with those measured by WES (R^2^=0.9869, **Figure 3A**) in the Giannakis dataset. Notably, the 102-exon signature with ~0.13 Mbp of coding genome was much shorter than the MSK-IMPACT (~0.92 Mbp) and FoundationOne gene panel (~1.1 Mbp), which may be a more cost-effective solution for the TMB estimation of blood and/or biopsy specimens.

In order to test the performances of the 102-exon signature, we applied it to three independent validation datasets. Compared to WES TMB, the relative number of identified mutations by the 102-exon signature was lower as indicated by a more gentle slope of the linear regression compared to the expected correlation plot. For the 225 RCC samples in TCGA dataset and 57 RCC samples in Vasaikar dataset, the correlations of the TMB detected by the 102-exon signature and WES TMB were R^2^=0.9351 (**Figure 3B**) and R^2^=0.8063 (**Figure 3C**), respectively. A similar result was observed in the Seshagiri dataset with 72 colorectal cancer samples (R^2^=0.9527, **Figure 3D**), indicating that the 102-exon signature is precise for estimating TMB. What's more, we also found that the performances of the 102-exon signature were quite similar to the MSK-IMPACT and FoundationOne gene panel.

We next further determined whether the performance of the 102-exon signature was superior to randomly selected panels with 102 exons. After 1000 times stratified randomized resampling procedure, we found that the R^2^ of our signature was shown to be far higher than all R^2^ measured by randomly selected exon models in the Giannakis dataset (**Figure 3E**). Meanwhile, similar results were obtained in the TCGA, Vasaikar and Seshagiri datasets (**Figure 3F-H**), which demonstrated that the exon signature outperforms than expected by chance.

### Function analysis of the genes within 102-exon signature

The 102 exons of the signature are contained in 101 genes, among which many genes are cancer driver genes and some may contribute to the accumulation of somatic mutations. For instance, *RNF43*, *PTCH1*, *MN1* and* MDM2* are known as oncogenes or tumor suppressor genes documented in the Catalogue of Somatic Mutations (COSMIC, version 89, released on May 15, 2019) database 39. Activated *DPYD* is crucial to enhance the repair of DNA double-strand breaks to maintain euploidy 40. Similarly, *MUC16* mutations are associated with immune response and DNA replication and repair pathways 41, 42, implicating that MUC16 mutations may affect TMB and guide immunotherapy treatment 42.* NOS3*, *TTI1*, *RPS6KA3* and *ATP6V1B1* are mapped to PI3K-Akt-mTOR pathway. This pathway can regulate PD-L1 and inhibition of it may enhance CD8^+^ T cell infiltration within tumor tissue, resulting in reduced tumor burden 43. Additionally, some genes involved in our signature, such as *TAP1* and *NGF*, are linked to immunodeficiency 44, 45, whose mutations may destroy the immune system and drive the aberrant mutation accumulation in somatic cells. While some other genes are not well-recognized and the correlation of them and TMB may merit further investigation.

Taken together, many genes within our signature can contribute to somatic mutation burden.

### Comparison with an existing colorectal-specific TMB estimation model

**Figure 4:**
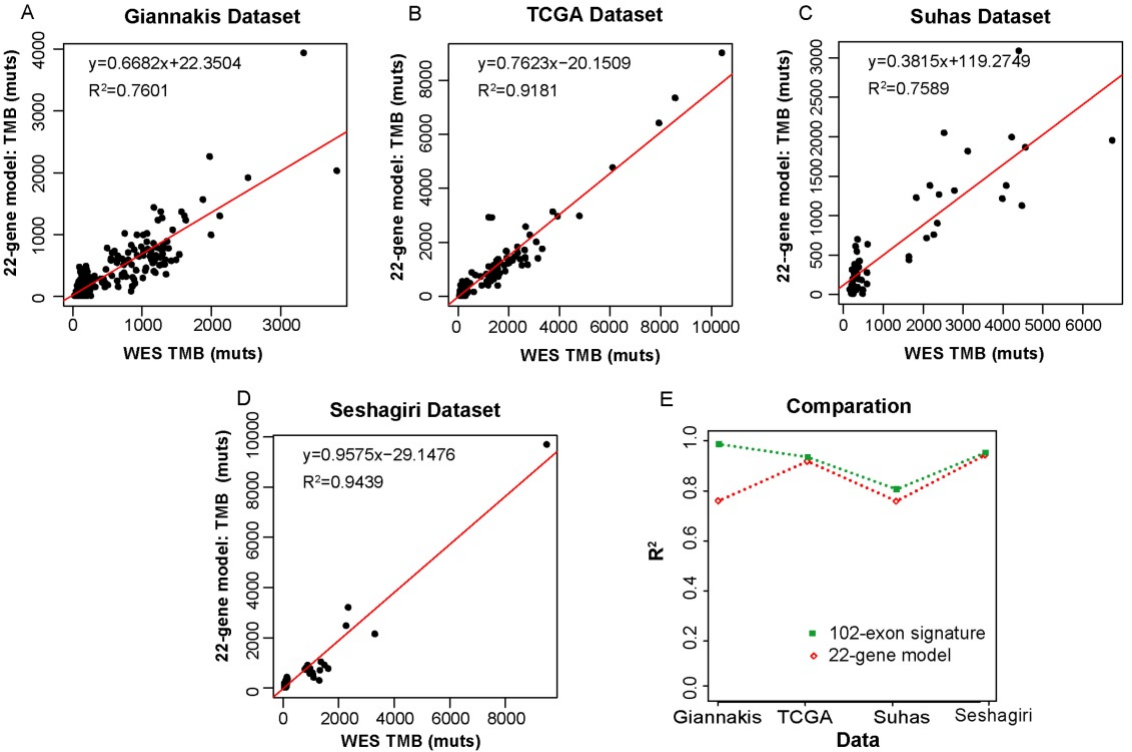
** Correlation of the TMB measured by the 22-gene model and WES TMB**. (A-D) WES TMB versus estimated TMB measured by the 22-gene model in Giannakis (A), TCGA (B), Vasaikar (C) and Seshagiri (D) dataste, respectively. (E) The performance comparison between the 102-exon signature and the 22-gene model.

We then compared our 102-exon signature with a colorectal-specific TMB estimation model 28, which is composed of 22 genes (~0.24 Mbp) and their corresponding parameters. We applied the 22-gene model to the four datasets as described above. In the Giannakis dataset with 315 RCC samples, R^2^ between the estimated TMB and WES TMB was shown to be 0.7601 (**Figure 4A**). Similarly, the TMB estimated by the 22-gene model was moderately correlated with that assessed by WES in the Vasaikar dataset (R^2^=0.7586) (**Figure 4C**). Whereas in the TCGA dataset, which was used as the training data to construct the 22-gene model, the correlation between the estimated TMB and WES TMB was far increased (R^2^=0.9181, **Figure 4B**). And a strong correlation was also observed in the Seshagiri dataset (R^2^=0.9439, **Figure 4D**). But compare to our 102-exon signature, the performances of the 22-gene model were much worse **(Figure 4E)**, especially in Giannakis and Vasaikar datasets. Moreover, without complex parameters, our exon signature may be more convenient and have higher clinical applicability in comparison with the 22-gene model.

### Molecular differences between TMB-high and TMB-low samples

In order to investigate the molecular characteristics of patients with different TMB levels, the tumors were divided into two groups according to the TMB discriminated threshold of 20 mut/Mbp of sequenced DNA 17, 46-48 (Table 1). After normalized against mutations per megabase, the range of TMB as detected by 102-exon signature was 0-252.75 mut/Mbp (Giannakis dataset), 0-380.15 mut/Mbp (TCGA dataset) and 0-289.92 mut/Mbp (Seshagiri dataset). The mean TMB values for the two groups in Giannakis dataset were 3.25 mut/Mbp (<20 mut/Mbp, TMB-low) and 62.95 mut/Mbp (≥20 mut/Mbp, TMB-high). And statistically significant was observed between the two groups (Wilcoxon rank sum test, *p*<2.2E-16). Similarly, differences were significant beween TMB-low and TMB-high groups in the TCGA (4.48 mut/Mbp vs 75.37 mut/Mbp), Vasaikar (2.89 mut/Mbp vs 116.11 mut/Mbp) and Seshagiri (1.70 mut/Mbp vs 63.93 mut/Mbp) datasets.

**Figure 5:**
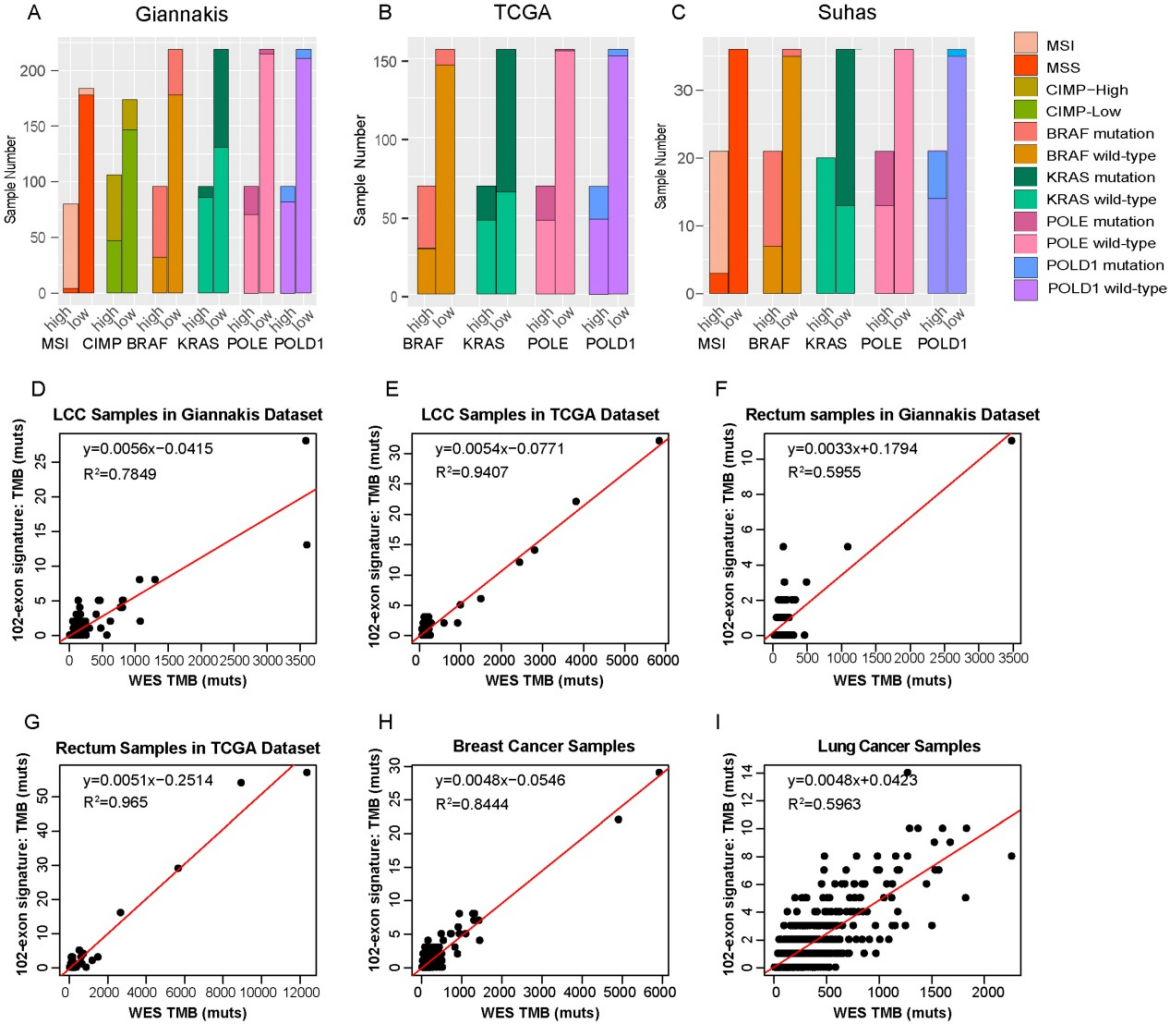
** Molecular differences between TMB-high and TMB-low groups and RCC-specific for the exon signature.** (A-C) The distributions of MSI, CIMP, BRAF, KRAS, POLE and POLD1 between TMB-high and TMB-low samples in Giannakis (A), TCGA (B) and Vasaikar(C) dataset. (D) and (E) WES TMB versus estimated TMB of the LCC patients in Giannakis (D), TCGA (E) dataset, respectively. (F) and (G) WES TMB versus estimated TMB in the rectum patients of Giannakis (F) and TCGA dataset, respectively. (H) and (I) WES TMB versus estimated TMB in breast cancer cohort and lung cancer cohort, respectively. high, TMB-high group; low, TMB-low group.

In line with previous studies 4-8, we observed that the TMB-high groups have significantly higher prevalences of MSI status and CIMP phenotype than the TMB-low groups (Fisher's exact test, *p*<0.01) in the RCC datasets. And tumors with TMB-high exhibited higher *BRAF* and lower* KRAS* mutation rate than the TMB-low patients as well. Additionally, we also found that TMB-high patients were enriched for defects in two mismatch repair pathway genes, *POLE* and *POLD1* (Table 2 and **Figure 5A-C**), which was consistent with the study reported by Campbell et al. 49.

### Cancer-specific for the exon signature

In order to investigate whether the 102-exon signature developed from RCC patients can also be employed to estimate TMB for patients with other cancer types, we firstly applied it to two cohorts of LCC samples. In the Giannakis dataset with 166 LCC patients, R^2^ between the estimated TMB with the WES TMB was shown to be 0.7849 (**Figure 5D**). In the TCGA dataset with 150 LCC patients, the linear correlation was shown to be 0.9407 (**Figure 5E**).

Then, the 102-exon signature was further applied to rectum, breast cancer and lung cancer patients, respectively. We observed that R^2^ between the estimated TMB and WES TMB were 0.5955 **(Figure 5F)** and 0.965 (**Figure 5G**) for the LCC samples in the Giannakis (n=137) and TCGA (n=137) dataset, respectively. In the breast cancer cohort with 986 samples and lung cohort with 567 patients, we also found that the linear correlations dramatically decreased (breast cancer, R^2^=0.8444, **Figure 5H**; lung cancer, R^2^=0.5963, **Figure 5I)**, indicating that a cancer-specific TMB estimated signature for a specific cancer type is necessary.

## Discussion

To the best of our knowledge, almost all of gene-based panels, which are developed to estimate TMB, are derived from multiple types of tumor patients. In this study, through a stratified randomized resampling procedure, we firstly demonstrated two non-cancer-specific and widely used gene panels (MSK-IMPACT and FoundationOne panels) performed similarly to randomly selected panels with the same number of genes, which suggested that those panels should be further optimized. Then, given that the considerable differences in mutational landscapes among different types of cancer and many of the exons of a gene are irrelevant with cancer, we directly selected a singature from the coding DNA sequences to estimate TMB for RCC patients. The exon signature allowed the precise estimation of the TMB in three independent WES RCC datasets and performed better than randomly selected panels with the same number of exons. Also, it has better performances than a colorectal-specific mutational burden estimation model contained 22 genes. The exon signature has only a total 134,522 bases, which is much shorter than that in the the commercial or institutional gene panels 23, 26, 50. Therefore, it can considerably decrease the cost and time required for the assessment of TMB, which will further accelerate the establishment of diagnosis and medical decisions.

When we applied the 102-exon signature to other types of cancer patients, its performance was dramatically degraded. Since there are considerable differences in the mutational landscapes between different types of cancer, a cancer-specific TMB estimation signature was shown to be necessary to estimate precisely the TMB in a specific type of cancer. Therefore, using the same methods as the one used for the RCC patients, we then utilized the somatic mutational data of LCC patients obtained from the Giannakis dataset (n=166) to train a LCC TMB estimated signature. The constructed LCC signature contained eight exons of eight genes (a total length of 10,641 bases). And R^2^ between the estimated TMB and WES TMB was 0.9691. However, when we applied it to 150 LCC patients in the TCGA dataset, R^2^ between the estimated TMB and WES TMB was only 0.5472. Indeed, there were few samples with high mutation rate in LCC patients (Supplementary Figure 1), which resulted in overfitting and a small number of exons could reach an optimal R^2^ value when we identified the LCC signature. To solve this issue, we could set a threshold for the number of exon regions when we develop the signature, which will be further studied in the follow-up work.

To date, there is no common ground which mutation types and regions should be included, and including all mutations instead of only missense mutations in the calculation of TMB has been debated 23, 24, 26, 27, 36, 51. Therefore, we analyzed if the number of other mutations is proportional to the number of missense mutations in the Giannakis, TCGA and Seshagiri datasets. For the Giannakis dataset, the linear correlation between non-missense and missense mutations was only 0.6567, which may be resulted from few non-missense mutations. But in the TCGA and Seshagiri dataset, higher correlations were observed (TCGA, R^2^=0.9978; Seshagiri, R^2^=0.9948), which indicated that include all point mutations in the calculation of TMB may even enhance the precision of the estimation to some extent. And there also is no agreed on the objective cut-points for TMB, so we defined TMB-high as ≥17 mut/Mbp 29, 52 and ≥12 mut/Mbp 4, 53 to investigate the molecular characteristics of patients with different TMB levels. Unexpectedly, similar molecular differences were found between the TMB-high and TMB-low group (Supplementary Table S2 and Table S3).

We certainly acknowledge that our study have several limitations. For example, the R^2^ values of our 102-exon signature were slightly lower than that of the MSK-IMPACT and FoundationOne gene panel in the Vasaikar, TCGA and Seshagiri datasets. It may be attributed to that our analysis did not include the differences of wet-lab factors that will influence TMB measurement between datasets, such as DNA quality and quantity, coverage and read depth of sequencing platforms and so on, which contributed to 10, 45 and 49 of the 102 exons were without a mutation in the the TCGA, Vasaikar and Seshagiri datasets, respectively. Furthermore, the immunotherapy response data for these colon cancer samples was not available, the treatment response prediction accuracy of the exon signature cannot be evaluated. Therefore, the performance of predicting the immunotherapy treatment response for RCC patients need to be delineated in future studies.

In summary, we have successfully constructed a mathematical signature using only 102 coding exons (~0.13 Mbp) that can be used to estimate the TMB in RCC samples precisely. The signature was much shorter than the existing gene panels, which could make the cost and time needed for the assessment of the TMB considerably decrease. Therefore, a customized panel for the targeted sequencing of these selected genes can be designed, instead of whole-exome sequencing.

## Supplementary Material

Supplementary figures and tables.

## Figures and Tables

**Table 1:** Description of whole-exome sequencing mutational data analyzed in this study

Cancer type			Datasets			
			TCGA	Giannakis	Vasaikar	Seshagiri
Colorectal cancer	Sample_number		512	618	57	72
	Location	Right	225	315	57	-
		Left	150	166	-	-
		Rectum	137	137	-	-
	MSI status of RCC samples	MSS	-	182	39	-
		MSI	-	82	18	-
		NA	-	51	-	-
Breast cancer	Sample_number		986	-		-
Lung cancer	Sample_number		537	-		-

Note: the Vasaikar dataset only include missense mutations.

**Table 2:** Molecular differences between TMB-high and TMB-low patients

Giannakis dataset			TMB-low (N=219)	TMB-high (N=96)	*p* value
	MSI*	MSI	6	76	<2.2E-16
		MSS	178	4	
		NA	35	16	
	CIMP*	CIMP_High	27	59	<2.2E-16
		CIMP_Low	147	17	
		NA	45	20	
	BRAF	mutation	41	64	3.64E-16
		wild-type	178	32	
	KRAS	mutation	88	86	3.76E-08
		wild-type	131	10	
	POLE	mutation	4	25	8.73E-11
		wild-type	215	71	
	POLD1	mutation	8	14	1.12E-03
		wild-type	211	82	
**TCGA dataset**			**TMB-low (N=156)**	**TMB-high (N=69)**	
	BRAF	mutation	10	40	<2.2E-16
		wild-type	146	29	
	KRAS	mutation	91	22	2.90E-04
		wild-type	65	47	
	POLE	mutation	1	22	6.12E-12
		wild-type	155	47	
	POLD1	mutation	4	21	6.59E-09
		wild-type	152	48	
**Vasaikar dataset TMB-low TMB-high** ** (N=36) (N=21)**					
	MSI	MSI	0	18	4.29E-12
		MSS	36	3	
	BRAF	mutation	1	14	1.92E-07
		wild-type	35	7	
	KRAS	mutation	23	1	6.97E-06
		wild-type	13	20	
	POLE	mutation	0	8	1.23E-04
		wild-type	36	13	
	POLD1	mutation	1	7	
		wild-type	35	14	2.66E-03

Note: *A sample was assigned to NA group if its MSI status (or CIMP phenotype) information was not obtained in the Giannakis dataset. The MSI and CIMP phenotype information were not available in the TCGA dataset. And the Vasaikar dataset missed the CIMP phenotype information as well. The Seshagiri dataset was not used to perform the molecular differences because of the lack of specific location information. *p* value was calculated by a two-tailed Fisher's exact test.

## Abbreviations

TMB: tumor mutational burden
MSI: microsatellite instability
RCC: right-sided colon cancer
LCC: left-sided colon cancer
WES: whole-exome sequencing
CDS: coding DNA sequences
