# Supplementary material for: An Exon Signature to Estimate the Tumor Mutational Burden of Right-sided Colon Cancer Patients
Source: J Cancer. 2020 Jan 1;11(4):883–92. doi: 10.7150/jca.34363 (PMC6959028; doi:10.7150/jca.34363)
Supplement: Supplementary file 1 — Supplementary figures and tables. [file jcav11p0883s1.pdf]

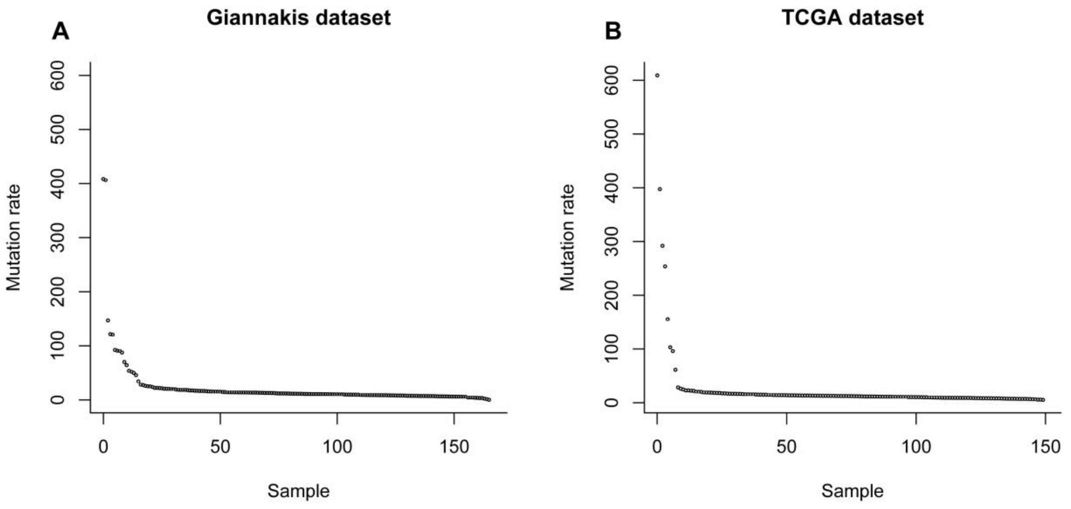

Figure S1. Mutational frequencies in the LCC samples. The mutational rate of left-sided colon cancer patients in the Giannakis (A) and TCGA (B) dataset.

Table S1. The compositions of the 102-exon signature.

gene	chrom	cdsStart	cdsEnd
MUC16	chr19	9056172	9077865
PLEC	chr8	144990344	144996563
GPR179	chr17	36482347	36487414
PLEKHA6	chr1	204228385	204228868
PCDHGA7	chr5	140762466	140764890
HIC1	chr17	1959964	1962129
FZD8	chr10	35928272	35930357
MN1	chr12	28192750	28196531
MAP1B	chr5	71489692	71496194
CSPG4	chr15	75979616	75983153
CMYA5	chr5	79024737	79035226
LUZP1	chr1	23417682	23420754
FCGBP	chr19	40362717	40363291
ZC3H13	chr13	46542882	46544106
CELSR2	chr1	109792701	109796011
JARID2	chr6	15496362	15497401
ZNF624	chr17	16525601	16527823
FBXL18	chr7	5540118	5541662
ARHGEF17	chr11	73019683	73022875
OR2C3	chr1	247694850	247695813

MDM2	chr12	69233053	69233629
HNRNPH2	chrX	100666976	100668326
PQLC1	chr18	77663975	77664183
SCG2	chr2	224462146	224464000
MYO9A	chr15	72189801	72191373
NGRN	chr15	90808944	90809108
OR51L1	chr11	5020212	5021160
MYH7B	chr20	33588095	33588492
PGBD2	chr1	249210800	249212562
PROX1	chr1	214169878	214171603
TTK	chr6	80720530	80720674
ZNF175	chr19	52089879	52091720
WBP1	chr2	74687347	74687808
TOR4A	chr9	140173141	140174413
HECTD1	chr14	31604077	31604401
SPECC1L	chr22	24717255	24718886
TP53BP2	chr1	224001941	224002055
NOS3	chr7	150698318	150698513
ZNF527	chr19	37879207	37880781
CAMSAP2	chr1	200826881	200827187
GPR146	chr7	1097151	1098153
ITIH5	chr10	7679190	7679441
SLC22A16	chr6	110763446	110763978
ABHD15	chr17	27889578	27890104
PPP4R1	chr18	9559416	9559602
DNAJA2	chr16	47005260	47005484
TMC1	chr9	75441784	75441910
ALDH1A3	chr15	101438287	101438390
OR4B1	chr11	48238361	48239291
TRIB1	chr8	126448247	126448713
PODXL2	chr3	127379220	127380002
RASSF9	chr12	86198479	86199740
GPRC5C	chr17	72435883	72436966
RNF43	chr17	56439904	56440009
POFUT2	chr21	46687504	46687628
PTCH1	chr9	98209193	98209733
FRMD4A	chr10	13701322	13701490
DNAH7	chr2	196728872	196729745
TTI1	chr20	36627589	36627730
HTRA3	chr4	8288287	8288510

HCFC1	chrX	153219516	153220993
AOC1	chr7	150553558	150555128
SOGA3	chr6	127836658	127837759
TAP1	chr6	32820815	32821593
PLCXD2	chr3	111432733	111432975
ATXN1	chr6	16306559	16307090
B4GALNT1	chr12	58021904	58022045
CAND2	chr3	12857872	12859375
TOX	chr8	59764082	59764364
MYO3B	chr2	171509492	171509631
TJP2	chr9	71835802	71836412
ZNF485	chr10	44111738	44112817
RSPH6A	chr19	46317784	46318434
SYNE1	chr6	152527302	152527497
C17orf97	chr17	262938	263906
ARSJ	chr4	114823429	114824831
RNF43	chr17	56492686	56492938
EPB41L5	chr2	120836068	120836157
SEMA3F	chr3	50220336	50220451
ILDR2	chr1	166889943	166890616
SLPI	chr20	43883099	43883184
RNF169	chr11	74546590	74547775
ATP6V1B1	chr2	71191567	71191672
DPYD	chr1	97771732	97771853
BRWD3	chrX	79932107	79932862
EGLN2	chr19	41306477	41307320
NGF	chr1	115828690	115829416
DVL2	chr17	7129183	7129632
OR6Q1	chr11	57798424	57799378
ATG9A	chr2	220088827	220089576
OR10A7	chr12	55614808	55615759
GPR153	chr1	6314609	6314965
GORASP1	chr3	39142506	39142593
SEPT9	chr17	75398140	75398785
TJP3	chr19	3738894	3739132
NRXN2	chr11	64374667	64375554
ZEB2	chr2	145161482	145161697
SLC22A11	chr11	64323471	64323864
MID2	chrX	107083899	107084615
DENND4B	chr1	153916533	153916850

SENP1	chr12	48458847	48459003
RPS6KA3	chrX	20205945	20206088

Note: the cdsStart denoted the start point of the CDS region; the cdsEnd denoted the end point of the CDS region.

Table S2. Molecular differences between TMB-high and TMB-low patients

Giannakis dataset		TMB-low (N=219)	TMB-high (N=96)	*p*
MSI*	MSI	6	76	<2.2E-16
	MSS	178	4	
	NA	35	16	
CIMP*	CIMP_High	27	59	<2.2E-16
	CIMP_Low	147	17	
	NA	45	20	
BRAF	mutation	41	64	3.64E-16
	wild-type	178	32	
KRAS	mutation	88	86	3.76E-08
	wild-type	131	10	
POLE	mutation	4	25	8.73E-11
	wild-type	215	71	
POLD1	Mutation	8	14	1.12E-03
	wild-type	211	82	
TCGA		TMB-low (N=156)	TMB-high (N=69)	
BRAF	mutation	10	40	<2.2E-16
	wild-type	146	29	
KRAS	mutation	91	22	2.90E-04
	wild-type	65	47	
POLE	mutation	1	22	6.12E-12
	wild-type	155	47	

POLD1	mutation	4	21	6.59E-09
	wild-type	152	48	
Suhas dataset		TMB-low (N=36)	TMB-high (N=21)	
MSI	MSI	0	18	4.29E-12
	MSS	36	3	
BRAF	mutation	1	14	1.92E-07
	wild-type	35	7	
KRAS	mutation	23	1	6.97E-06
	wild-type	13	20	
POLE	mutation	0	8	1.23E-04
	wild-type	36	13	
POLD1	mutation	1	7	
	wild-type	35	14	2.66E-03

Note: *The MSI status information for 51 RCC samples and CIMP phenotype information for 65 RCC samples were not available in the Giannakis dataset. There were no MSI and CIMP status information in the TCGA datasets. And the Suhas dataset missed the CIMP phenotype information as well. TMB discriminated threshold was 17 mut/Mb. *p* was calculated by Fisher's exact test.

Table S3. Molecular differences between TMB-high and TMB-low patients

Giannakis dataset		TMB-low (N=210)	TMB-high (N=105)	*p*
MSI*	MSI	5	77	<2.2E-16
	MSS	171	11	
	NA	34	17	
CIMP*	CIMP_High	25	61	<2.2E-16
	CIMP_Low	142	22	
	NA	43	22	
BRAF	mutation	36	69	<2.2E-16

		wild-type	174	36	
	KRAS	mutation	87	11	6.83E-09
		wild-type	123	94	
	POLE	mutation	4	25	9.64E-10
		wild-type	206	80	
	POLD1	mutation	7	15	6.63E-04
		wild-type	203	90	
TCGA			TMB-low (N=139)	TMB-high (N=86)	
	BRAF	mutation	10	40	9.05E-12
		wild-type	129	46	
	KRAS	mutation	82	31	9.82E-04
		wild-type	57	55	
	POLE	mutation	1	22	1.58E-09
		wild-type	138	64	
	POLD1	mutation	3	22	7.69E-08
		wild-type	136	64	
Suhas dataset			TMB-low (N=32)	TMB-high (N=25)	
	MSI	MSI	0	18	1.55E-09
		MSS	32	7	
	BRAF	mutation	1	14	3.88E-06
		wild-type	33	11	
	KRAS	mutation	20	4	4.84E-04
		wild-type	12	21	
	POLE	mutation	0	8	5.64E-04
		wild-type	33	17	
	POLD1	mutation	1	7	0.0168
		wild-type	32	18	

Note: *A sample was assigned to NA group if its MSI status (or CIMP phenotype) information was not obtained in the Giannakis dataset. There were no MSI and CIMP status information in the TCGA datasets. The Suhas dataset did not provide the CIMP phenotype information as well. TMB discriminated threshold was 12 mut/Mb. *p* was calculated by Fisher's exact test.
